# Antimicrobial and Antibiofilm Activity of Chitosan on the Oral Pathogen *Candida albicans*

**DOI:** 10.3390/pathogens3040908

**Published:** 2014-12-11

**Authors:** Eduardo Costa, Sara Silva, Freni Tavaria, Manuela Pintado

**Affiliations:** Universidade Católica Portuguesa/Porto, Rua Arquiteto Lobão Vital, Apartado 2511, 4202-401 Porto, Portugal; E-Mails: emcosta@porto.ucp.pt (E.C.); snsilva@porto.ucp.pt (S.S.); ftavaria@porto.ucp.pt (F.T.)

**Keywords:** candidiasis, *Candida albicans*, chitosan, biofilm, antibiofilm

## Abstract

Oral candidiasis is particularly evident, not only in cancer patients receiving chemotherapy, but also in elderly people with xerostomy. In general, *Candida* is an opportunistic pathogen, causing infections in immunocompromised people and, in some cases, when the natural microbiota is altered. Chitosan, a natural derivative of chitin, is a polysaccharide that has been proven to possess a broad spectrum of antimicrobial activity that encompasses action against fungi, yeast and bacteria. While recent studies have revealed a significant antibiofilm activity upon several microorganisms, including *C. albicans*, little is known regarding the impact of chitosan upon the adhesive process or mature biofilms. With that in mind, the purpose of this work was to evaluate, *in vitro*, the capability of chitosan to inhibit *C. albicans* growth and biofilm formation. The results obtained showed that chitosan is capable of inhibiting *C. albicans* planktonic growth (HMW, 1 mg/mL; LMW, 3 mg/mL). Regarding biofilm growth, chitosan inhibited *C. albicans* adhesion (*ca*. 95%), biofilm formation (percentages above 90%) and reduced mature biofilms by *ca.* 65% and dual species biofilms (*C. albicans* and *S. mutans*) by *ca.* 70%. These results display the potential of this molecule to be used as an effective anti-*Candida* agent capable of acting upon *C. albicans* infections.

## 1. Introduction

Since the 1970s, there has been an increase in candidiasis incidence, mostly due to the use of plastic permanent catheters, antibiotics and immunosuppressive drugs [1]. These *Candida*-derived infections may occur in the skin, mucous membranes (such as the mouth and vagina) and in the viscera, with the main etiological agent being *Candida albicans* [1,2]. Among the various human fungal pathogens, *C. albicans* accounts for the majority of systemic infections in immunocompromised patients, with overall mortality rates ranging from 29% to 76% [2,3,4,5,6]. This opportunistic fungi causes great problems, as it is resistant to most antimicrobial compounds, namely amphotericin-B, which is considered the standard for the treatment of systemic mycoses. Despite still being considered the drug of choice against *C. albicans*, these antifungal agents are being increasingly reported as inefficient with numerous cases of resistances, particularly to fluconazole, being observed [1,2,3,4]. This problem has led to the search for alternative drugs and compounds to be used in the treatment and management of *C. albicans* infections.

Chitin is the primary structural component of the shells of crustaceans, arthropods and the fungal cell wall and is obtained mainly as a byproduct of the fishing industry. Partial deacetylation of chitin leads to chitosan, a polysaccharide composed of units of glucosamine (2-amino-2-deoxy-d-glucose) and *N*-acetyl glucosamine (2-acetamido-2-deoxy-d–glucose) linked by β(1→4) bonds. Chitosan is the only natural polysaccharide that presents a cationic character due to its amino groups, which, at low pH, are protonated and can interact with negatively-charged compounds, such as proteins, anionic polysaccharides (e.g., alginates, carraghenates, pectins), fatty acids, bile acids and phospholipids [7]. This behavior, along with its biocompatibility, biodegradability and lack of toxicity, has led to the usage of chitosan in diverse fields, such as technology, food, cosmetics, medicine, biotechnology, agriculture and the paper industry [8,9]. However, chitosan possesses some limitations, namely its insolubility in water, high viscosity and tendency to coagulate proteins at high pH [10,11,12].

Chitosan’s antimicrobial activity is well established against a variety of microorganisms, including fungi [10,13,14,15]. When considering chitosan antifungal activity, several authors have already shown that it is active upon yeasts, molds and dermatophytes [16,17,18,19]. While the antifungal activity of chitosan upon *C. albicans* is well established, the same cannot be said regarding the effect of chitosan upon *C. albicans* biofilm formation. Early reports [20,21,22] suggest that chitosan may be active upon *C. albicans* biofilms; however, the real effect of chitosan upon the different steps of *C. albicans* biofilms has not yet been fully explored. As such, the aim of this work was to fully assess chitosan’s potential as a means to prevent *C. albicans*-derived infections through the control of its growth, adhesion and biofilm formation.

## 2. Results and Discussion

### 2.1. MIC Determination

The MIC values, obtained by broth microdilution, for chitosan activity upon *C. albicans* were relatively low. In fact, HMW chitosan presented a MIC value of 1 mg/mL and LMW chitosan a MIC value of 3 mg/mL. The antifungal activity of chitosan upon *C. albicans* is well established, with several authors [16,17,18,19] presenting various MIC values for different chitosans against this yeast. Tayel, Moussa, El-Tras, Knittel, Opwis and Schollmeyer [2] previously reported a MIC of 1.25 mg/mL (32 kDa, deacetylation degree (DD) 86%). Qin *et al.* [23] reported an even lower MIC of 0.8 mg/mL (2.91 kDa, DD 86.4%), and Şenel *et al.* [24] reported a MIC of 10 mg/mL (1,000 kDa, DD 80%). Comparing these results with the ones obtained, it is possible to see that for LMW chitosan, the MIC value obtained was slightly superior to those previously reported [2,23], with this differences being probably due to the higher DD used in those assays. On the other hand, for HMW chitosan, the values here obtained were significantly lower than those reported by Şenel, İkinci, Kaş, Yousefi-Rad, Sargon and Hıncal [24]. From here, the ½ and the ¼ of the MIC were calculated to be used in the biofilm assays, as previously described by Cerca *et al.* [25].

### 2.2. Adherence to Coated Surfaces

**Figure 1:**
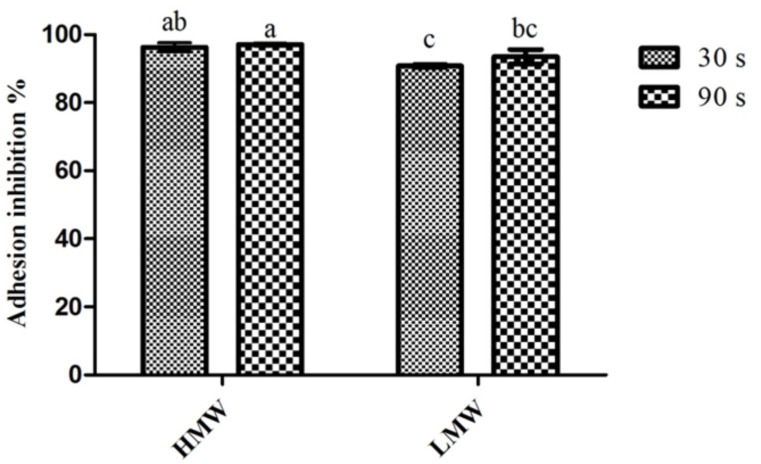
Inhibitory effect of chitosan upon *C. albicans* adhesion. Values obtained given as the percentage of adhesion inhibition. Different letters represent the statistically significant differences found (*p* < 0.05). All assays performed in triplicate. HMW, high molecular weight; LMW, low molecular weight.

The effect of chitosan upon *C. albicans* adhesion to surfaces can be seen in Figure 1. The results obtained showed that both MW and the times tested were capable of producing adhesion inhibition percentages above 90%. In fact, the lowest inhibition percentage was obtained for LMW chitosan after only 30 s of exposure. When considering the differences between 30 s and 90 s of exposure, there were no significant statistical differences (*p* > 0.05) found, either for HMW or LMW chitosan. On the other hand, when considering the impact of the MW and the exposure time, some differences are ascertainable; 90 s of exposure for HMW presented statistically significant (*p* < 0.05) higher inhibition values than both LMW assays; LMW, at 30 s of exposure, presented a significantly lower (*p* < 0.05) inhibition value than the one registered in both HMW assays. These results are in line with those previously reported by Carlson, Taffs, Davison and Stewart [20], who showed that chitosan reduced *C. albicans* adhesion up to 99%.

### 2.3. Microtiter-Plate Test

**Figure 2:**
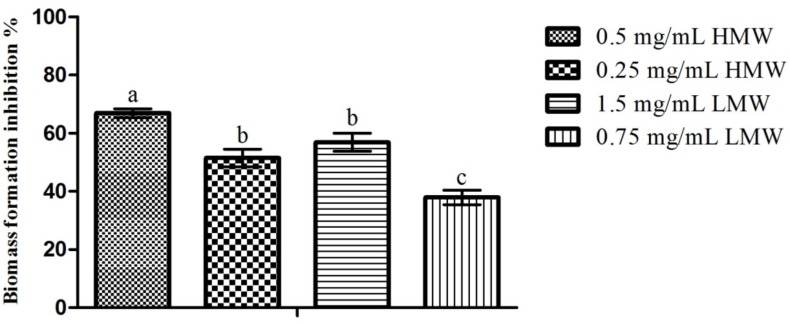
Effect of sub-MIC concentrations of chitosan (½ and ¼ of the MICs; values in mg/mL) upon *C. albicans* biofilm formation. Values obtained are given as the percentage of biofilm formation inhibition. Different letters represent the statistically significant differences found (*p* < 0.05). All assays were performed in triplicate.

When considering the impact of chitosan upon *C. albicans* biofilm formation (Figure 2), here analyzed indirectly through biomass production, one can see that, as with the previous assay, the highest inhibition percentage (66.94%) was obtained for HMW chitosan (0.5 mg/mL) and the lowest inhibition percentage (37.97%) was obtained for LMW chitosan (0.75 mg/mL). When comparing the results obtained for the ½ and ¼ of the MIC of both MWs, no statistically significant (*p* > 0.05) differences were found when considering the effect of the MW upon chitosan’s activity. On the other hand, when considering the effect of the MW in conjunction with the concentration, one can see clear differences in behavior (Figure 2). In fact, 0.5 mg/mL of HMW chitosan presented significantly higher (*p* < 0.05) inhibition values than the remaining assays, while no significant (*p* > 0.05) differences were found between the ¼ of the MIC for HMW (0.25 mg/mL) and ½ of the MIC for LMW (1.5 mg/mL). On the other hand, 0.75 mg/mL of LMW chitosan presented statistically significant (*p* < 0.05) lower inhibition percentages than the remaining assays. These results are in line with those registered by Martinez, Mihu, Tar, Cordero, Han, Friedman, Friedman and Nosanchuk [22], who reported that chitosan was capable of reducing *C. albicans* biofilm formation by a 2.5 factor, and by those of Cobrado *et al.* [26] and of Cobrado *et al.* [27], who showed that chitosan was capable of reducing *C. albicans* biomass production up to 90%.

### 2.4. Mature Biofilms Assays

**Figure 3:**
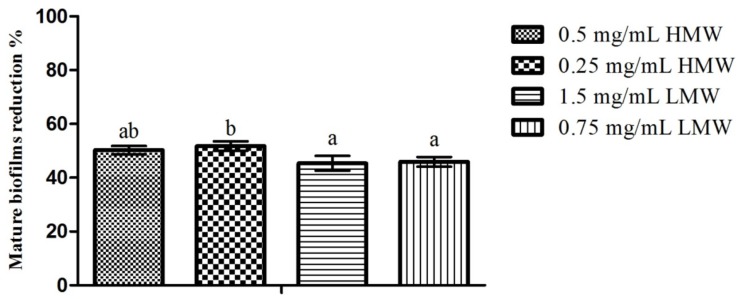
Effect of sub-MIC concentrations of chitosan (½ and ¼ of the MICs; values in mg/mL) upon *C. albicans* mature biofilms. Results are presented as biofilm reduction percentages. Different letters represent the statistically significant differences found (*p* < 0.05). All assays performed in triplicate.

Regarding the effect of chitosan upon *C. albicans* mature biofilms, the results obtained can be seen in Figure 3. Once again, the highest inhibition percentage was obtained for HMW chitosan (51.77% for 0.25 mg/mL), and the lowest inhibition was registered for LMW chitosan (45.37% for 1.5 mg/mL). Statistical analysis of the results showed that when considering the effect of the MW, there were statistically significant differences (*p* < 0.05) between HMW chitosan at 0.25 mg/mL and both LMW concentrations tested. Simultaneously, when considering the effect of the MW in conjunction with concentration (Figure 3), differences were also observed with 0.25 mg/mL HMW chitosan, presenting significantly (*p* < 0.05) higher inhibition values than the assays that utilized LMW chitosan. Between the remaining assays, no statistically significant (*p* > 0.05) differences were found.

### 2.5. Dual-Species Biofilms

**Figure 4:**
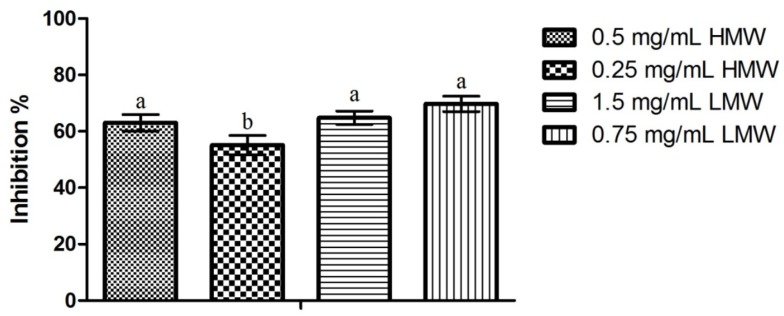
Effect of sub-MIC concentrations of chitosan (½ and ¼ of the MICs; values in mg/mL) upon biofilms formed by *C. albicans* and *S. mutans*. Results presented as the percentage of biofilm formation inhibition. Different letters represent the statistically significant differences found (*p* < 0.05). All assays performed in triplicate.

**Figure 5:**
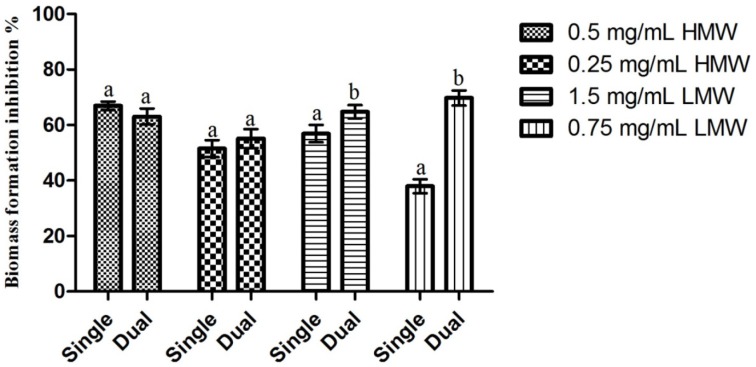
Comparison of the effect of sub-MIC concentrations of chitosan upon *C. albicans* single species and dual species biofilms. Results presented as the percentage of biofilm formation inhibition. Different letters represent the statistically significant differences found (*p* < 0.05). All assays performed in triplicate.

Results obtained regarding the activity of chitosan upon *C. albicans* mature biofilms can be observed in Figure 4. Contrary to the pattern observed in previous assays, LMW chitosan presented the highest biofilm inhibition percentage (66.77% for 0.75 mg/mL) and HMW chitosan the lowest (55.10% for 0.25 mg/mL). Statistical analysis of the results showed that the only statistically significant difference observed was for 0.25 mg/mL of HMW chitosan, which presented an inhibition value significantly lower than the inhibition values obtained in the remaining test conditions. When considering the differences in chitosan’s activity between single species *C. albicans* biofilm and dual species *C. albicans* and *S. mutans* biofilms (Figure 5), the statistical analysis shows that there are statistically significant differences (*p* < 0.05) in LMW chitosan’s activity between a single and a dual species population. On a closer look, LMW chitosan presents a statistically significant (*p* < 0.05) increase in activity between single species and dual species biofilms. This translates as an increase of biomass production inhibition of *ca*. 10%, for 1.5 mg/mL, and of *ca*. 31%, for 0.75 mg/mL of chitosan, for LMW chitosan between populations.

Despite the lack of previous results regarding the effect of chitosan upon *C. albicans* mature and dual species biofilms, the inhibitions here registered are quite interesting, especially when considering that *C. albicans* biofilms produce an exopolymeric matrix that serves as a diffusion barrier to antimicrobials and that, under these conditions, *Candida* cells overexpress efflux pumps to enhance antifungal resistance [28]. This mechanism may be the reason why HMW chitosan possessed higher activity than LMW upon mature biofilms, as it is known that the latter must enter the cells in order to be active [15]. In the dual species biofilms, one cannot underestimate the importance of *S. mutans*, as it is known to be crucial to *C. albicans* colonization of the oral cavity, mainly due to providing adhesion sites and producing lactate that can be used as a carbon source by yeasts. This symbiosis has been well established in several studies, which have shown that there is a strong coadherence between these microorganisms [29]. Considering that, both *S. mutans* and *C. albicans*, have been described as being more sensitive to HMW chitosan [16,17,18,19,30], it is somewhat surprising that LMW chitosan presented higher inhibition percentages than HMW chitosan for the dual species biofilms. It is possible that an unknown mechanism, possibly located at the adhesins level, as hypothesized by Azcurra *et al.* [31], or at the cell to cell communication level, where the larger HMW molecules are incapable of acting, is responsible for the higher activity registered for LMW chitosan.

## 3. Experimental Section

### 3.1. Sources of Chitosan and Microorganisms

High and low molecular weight chitosan were obtained from Sigma-Aldrich (St. Louis, MO, USA). High molecular weight chitosan was characterized by a DD > 75% and a MW of 624 kDa. Low molecular weight chitosan was characterized by a DD between 75% and 85% and a MW of 107 kDa. Chitosan solutions were prepared in 1% (v/v) solution of glacial acetic acid 99% (Panreac, Barcelona, Spain). Chitosan was added to 1% acetic acid to the desired concentration. Afterwards, the solution was stirred overnight at 50 °C to promote complete dissolution of chitosan. The pH was adjusted with NaOH (Merck, Darmstad, Germany) to a final value of 5.6–5.8, and solutions were stored at refrigerated temperature.

*Candida albicans* used in this study was obtained from the culture collection of the Göteburg University (CCUG) (Sweden) (CCUG 49242). Inocula were prepared in yeast malt broth (YMB) (Difco, Franklin Lakes, NJ, USA) and incubated at 37 °C for 24 h. Viable counts were performed in yeast malt agar (YMA) (Difco, Franklin Lakes, NJ, USA).

### 3.2. Determination of Minimal Inhibitory Concentration

Determination of the MIC was performed as described by Costa, Silva, Pina, Tavaria and Pintado [10]. Briefly, an inoculum of 0.5 in the MacFarland scale (1.5 × 10^8^ CFU/mL) of *C. albicans* was prepared from overnight cultures and inoculated in YMB with chitosan concentrations ranging from 0.1 mg/mL to 7 mg/mL. Two controls were simultaneously assessed: one with 0.1 mg/mL chitosan, but without inoculum, and another where chitosan was replaced by sterile water and with added inoculum. The MIC was determined by observing the lowest concentration of chitosan that inhibited microbial growth. All assays were performed in triplicate.

### 3.3. Adherence

The effect of chitosan on *C. albicans* adhesion to surfaces was performed as described by Costa, Silva, Tavaria and Pintado [30], tested using 24-well microplates. Briefly, 1 cm aluminum disks were dipped for 30 or 90 s in a well containing either 1% (v/v) HMW or LMW chitosan. Following that, the disks were rinsed with sterile water and submerged in a well containing inoculum for 60 s, after which disks were placed into wells containing the appropriate medium and incubated for 24 h at 37 °C. Two controls were simultaneously assessed. In the first one, disks were dipped in sterile water and then inoculated and incubated. In the second one, disks were dipped in the test solutions and, after rinsing in sterile water, were then incubated without inoculum. After 24 h, the disks were recovered, and after serial dilutions, viable counts were assessed by the drop method, as described by Miles *et al.* [32], in YMA. Plates were then incubated at 37 °C for 24 h under aerobic conditions. Results were given as inhibition percentages using the following formula:

% I = 100 − (log CFU sample/log CFU control) × 100

All assays were performed in duplicate.

### 3.4. Microtiter-Plate Test

Biofilm quantification was carried out by adapting the protocol of Stepanovic *et al.* [33]. Briefly, in a flat bottom, 96-well microplate, wells were filled with 200 µL of test solutions with chitosan added at sub-MIC concentrations (½ and ¼ of the MIC) and inoculated at 1% (v/v). The plate was then incubated at 37 °C for 24 h in aerobiosis. All assays were performed in triplicate in the appropriate media supplemented with 5% sucrose.

To visualize adhesion, the contents of each well were discarded and then washed 3 times with sterile deionized water in order to remove non-adherent cells. The remaining attached microorganisms were fixed with 200 µL of ethanol (Panreac, Barcelona, Spain) for 15 min. Ethanol was discarded, and the wells were air dried. After that, 200 µL of crystal violet solution were added to the wells for 5 min, the excess stain removed by rinsing the plate under tap water followed by air drying. Adherence was quantified by measuring the OD at 630 nm using a microplate reader (FLUOstar, OPTIMA, BGM Labtech).

Optical density values from wells with liquid media, chitosan and no inoculum were used as negative controls, while OD from wells with liquid media, deionized water and inoculum were used as positive controls. Additionally, a control with 1% (v/v) acetic acid for each microorganism was used.

Results for this test were given as the percentage of biofilm formation inhibition applying the following formula:

*% biofilm formation inhibition* = *100* − *(OD_assay_/OD_control_)* × *100*

### 3.5. Mature Biofilms Assay

The assessment of chitosan’s effect on mature biofilms was performed through adaptation of the microplate protocol described by Stepanovic, Vukovic, Dakic, Savic and Svabic-Vlahovic [33]. Briefly, in a flat bottom 96-well microplate, wells were filled with 200 µL of medium, inoculated at 1% (v/v) and incubated 48 h at 37 °C. After 48 h, the medium was carefully aspirated, and the wells were rinsed with phosphate buffer. Following that, 200 µL of medium, with chitosan at sub-MIC concentrations, was added and incubated at 37 °C for 24 h.

To visualize biofilms, the contents of each well were discarded and the wells washed 3 times with sterile deionized water in order to remove non-adherent cells. The remaining attached microorganisms were fixed with 200 µL of ethanol (Panreac, Barcelona, Spain) for 15 min. Ethanol was then discarded and the wells air dried. After that, 200 µL of crystal violet solution (Merck, Darmstadt, Germany) were added to the wells for 5 min. Excess stain was removed by rinsing the plate under tap water followed by air drying of the plate.

Adherence was quantified by measuring the OD at 660 nm using a microplate reader.

All experiments were done in triplicate for each microorganism. OD values from wells only with YMB were used as negative controls. A positive control with media and sterile deionized water was used. Additionally a control with 1% (v/v) acetic acid for each microorganism was used.

Results for this test were given as the reduction of the present biofilm, applying the following formula:

Mature biofilm inhibition percentage = 100 − (OD_assay_/OD_control_) × 100

### 3.6. Dual-Species Biofilms

Quantification of the effect of chitosan upon biofilms formed by two different microorganisms was performed as previously described by Costa, Silva, Tavaria and Pintado [30]. Briefly, a test solution, with chitosan at sub-MIC concentrations, was inoculated with *C. albicans* and *Streptococcus mutans* (CCUG 45091) (1:1) to achieve a 2% (v/v) inoculum concentration*.* Impact upon biofilm formation was evaluated using the biofilm microtiter plate assay as described above. Results were obtained as referred above, and all assays were done in triplicate.

### 3.7. Statistical Treatment

The statistical differences in the methods were evaluated using PASW Statistics v. 21.0.0.0 (New York, NY, USA). The normality of the results’ distribution was evaluated through Shapiro–Wilk’s test. The differences were assessed using the one-way ANOVA test associated with Scheffe’s test (for normal distributions). The differences were considered significant at a 0.05 significance level.

## 4. Conclusions

In conclusion, chitosan showed remarkable potential as a possible anti-candidiasis agent, as it was active upon *C. albicans* in the planktonic state and, more importantly, upon its sessile growth, with significant activity upon the several phases—adhesion, formation, mature and co-aggregation—of biofilm establishment and growth.
